# Autoimmune hepatitis displays distinctively high multi-antennary sialylation on plasma *N*-glycans compared to other liver diseases

**DOI:** 10.1186/s12967-024-05173-z

**Published:** 2024-05-14

**Authors:** Tamas Pongracz, Maaike Biewenga, Anna Eva Charlotte Stoelinga, Marco René Bladergroen, Simone Nicolardi, Leendert Adrianus Trouw, Manfred Wuhrer, Noortje de Haan, Bart van Hoek

**Affiliations:** 1 Center for Proteomics and Metabolomics, Leiden University Medical Center, Albinusdreef 2, Leiden, 2333 ZA The Netherlands; 2 Department of Gastroenterology and Hepatology, Leiden University Medical Center, Albinusdreef 2, Leiden, 2333 ZA The Netherlands; 3 Department Immunology, Leiden University Medical Center, Albinusdreef 2, Leiden, 2333 ZA The Netherlands

**Keywords:** Autoimmune hepatitis, Liver inflammation, Plasma *N*-glycosylation, IgG glycosylation, Tetraantennary glycans, Glycome, Biomarker

## Abstract

**Background:**

Changes in plasma protein glycosylation are known to functionally affect proteins and to associate with liver diseases, including cirrhosis and hepatocellular carcinoma. Autoimmune hepatitis (AIH) is a liver disease characterized by liver inflammation and raised serum levels of IgG, and is difficult to distinguish from other liver diseases. The aim of this study was to examine plasma and IgG-specific *N*-glycosylation in AIH and compare it with healthy controls and other liver diseases.

**Methods:**

In this cross-sectional cohort study, total plasma *N*-glycosylation and IgG Fc glycosylation analysis was performed by mass spectrometry for 66 AIH patients, 60 age- and sex-matched healthy controls, 31 primary biliary cholangitis patients, 10 primary sclerosing cholangitis patients, 30 non-alcoholic fatty liver disease patients and 74 patients with viral or alcoholic hepatitis. A total of 121 glycans were quantified per individual. Associations between glycosylation traits and AIH were investigated as compared to healthy controls and other liver diseases.

**Results:**

Glycan traits bisection (OR: 3.78 [1.88–9.35], *p*-value: 5.88 × 10^− 3^), tetraantennary sialylation per galactose (A4GS) (OR: 2.88 [1.75–5.16], *p*-value: 1.63 × 10^− 3^), IgG1 galactosylation (OR: 0.35 [0.2–0.58], *p*-value: 3.47 × 10^− 5^) and hybrid type glycans (OR: 2.73 [1.67–4.89], *p*-value: 2.31 × 10^− 3^) were found as discriminators between AIH and healthy controls. High A4GS differentiated AIH from other liver diseases, while bisection associated with cirrhosis severity.

**Conclusions:**

Compared to other liver diseases, AIH shows distinctively high A4GS levels in plasma, with potential implications on glycoprotein function and clearance. Plasma-derived glycosylation has potential to be used as a diagnostic marker for AIH in the future. This may alleviate the need for a liver biopsy at diagnosis. Glycosidic changes should be investigated further in longitudinal studies and may be used for diagnostic and monitoring purposes in the future.

**Supplementary Information:**

The online version contains supplementary material available at 10.1186/s12967-024-05173-z.

## Background

Autoimmune hepatitis (AIH) is a chronic autoimmune disease of the liver, characterized by autoantibodies and elevated circulating total immunoglobulin gamma (IgG) [1]. Diagnosis of AIH is based on a scoring system which combines, amongst others, autoantibodies, IgG level and the results of a liver biopsy [2, 3]. However, it can still be difficult to distinguish AIH from other (autoimmune) liver diseases such as primary biliary cholangitis (PBC), primary sclerosing cholangitis (PSC) or non-alcoholic fatty liver disease (NAFLD), of which prevalence is increasing. Additionally, a liver biopsy is an invasive procedure with a risk of bleeding. The addition of new, non-invasive diagnostic biomarkers in AIH would facilitate the diagnosis and might reduce the need for liver biopsy in the future.

The aim of the treatment of AIH is complete biochemical response (CBR), defined as normalization of aminotransferases and IgG. CBR is associated with a reduced disease progression and better long-term survival [4–6]. While a liver biopsy is informative in disease monitoring, repeated liver biopsies are associated with high risks for complications. In clinical practice disease activity is determined by aspartate transaminase (AST), alanine transaminase (ALT) and IgG level. In some patients only partial biochemical remission can be obtained and relapses or loss of remissions can occur especially after treatment is stopped [7]. More specific, blood-derived biomarkers would allow for a more tailored and less invasive monitoring of the patients.

The post-translational modification of proteins by *N*-glycosylation adds a layer of functional complexity to the majority of human proteins, with acknowledged immunological and homeostatic roles, as differences in *N*-glycan structures may influence the plasma half-life of glycoproteins as well as their functions [8, 9]. As a major part of the plasma glycoproteins is synthesized – and glycosylated – in the liver, liver disease-induced glycosylation changes of plasma glycoproteins are of high interest. Previously, specific glycosylation patterns have been associated with non-alcoholic steatohepatitis (NASH), chronic hepatitis C virus (HCV), fibrosis, but also cirrhosis and risk of hepatocellular carcinoma (HCC) [10–15]. The glycosylation pattern associated with HCC forms the basis of the clinically available GlycoCirrhoTest, specifically measuring the ratio between the proportion of bisecting *N*-acetylglucosamine-containing *N*-glycans and triantennary *N*-glycans on circulatory glycoproteins [10]. Distinct changes in plasma protein glycosylation have also been associated with several (autoimmune) diseases including rheumatoid arthritis (RA), inflammatory bowel disease (IBD) and type 2 diabetes mellitus [16–19].

IgG is a non-liver-derived plasma glycoprotein of which elevated levels are found in AIH [4]. The various effector functions of IgG are highly dependent on the glycosylation (the presence and constitution of various monosaccharides) of the fragment crystallizable (Fc) region in its constant domain. For example, fucosylation of the IgG Fc glycan limits IgG-mediated antibody-dependent cellular cytotoxicity by lowering the affinity of IgG for the FcγRIII receptor [20]. Furthermore, the degree of Fc glycan galactosylation is strongly associated with inflammation. This protein-specific glycan trait decreases with aging as well as in several (autoimmune) diseases, and dynamically relates to disease behaviour as exemplified in RA, IBD, HCV and also COVID-19 [13, 21–28]. With the known association between IgG Fc galactosylation and inflammation, we hypothesize that this relationship will also be demonstrated in patients with AIH, given both the autoimmune nature and the hepatic inflammation characterizing the disease.

Studies on glycosylation in autoimmune liver disease, and AIH in particular have hitherto been lacking. Based on the promising liver disease-related plasma glycosylation effects reported in previous studies and the key role for plasma IgG levels in AIH, in the current study we aimed to extensively explore global plasma protein *N*-glycosylation (the total plasma *N*-glycome, TPNG) as well as IgG-specific glycosylation, in the context of differential AIH diagnosis and disease monitoring. To study glycosylation, state-of-the-art mass spectrometry (MS)-based methods were used on patient plasma material from a retrospective cross-sectional cohort [29]. Plasma and IgG glycan levels were compared between AIH patients and healthy controls, as well as patients with PBC, PSC, NAFLD and viral or alcoholic hepatitis with different stages of cirrhosis, i.e., without cirrhosis, compensated cirrhosis or decompensated cirrhosis (WC, CC and DC, respectively). Special attention was paid to known confounders of glycosylation, including sex, age, cirrhosis, and the use of immunosuppressive medication. The primary aim of this study was to find markers (i.e., specific glycomic alterations) with diagnostic potential for AIH, reflected in the TPNG. The secondary aim was to analyse the influence of cirrhosis and disease activity on these potential novel biomarkers.

## Patients and methods

### Study design

In this cross-sectional study, samples were obtained from the Leiden University Medical Center biobank. All AIH patients were diagnosed according to the simplified [2] or revised [3] AIH criteria and samples between 2004 and 2020, before or during treatment, were included. Patients with variant syndromes (i.e., AIH-PBC and AIH-PSC variant syndrome) were excluded. Matched on age and sex to 66 AIH patients (with overall 214 samples), 60 healthy controls were included. Furthermore, as control groups, 31 PSC, 10 PBC and 30 NAFLD were included, as well as 15 patients with viral or alcoholic hepatitis without cirrhosis, 29 patients with compensated cirrhosis and 30 patients with decompensated cirrhosis. PSC, PBC and NAFLD were diagnosed in accordance with the available guidelines [30–32]. Viral hepatitis was diagnosed using conventional viral markers and alcoholic hepatitis was determined based on daily alcohol intake. No prior sample size calculation was performed, samples were included based on availability. Cohort characteristics can be found in Tables 1 and 2 and Supplementary Table 1.

**Table 1:** Baseline and longitudinal characteristics of patients and healthy controls enrolled in the cohort. Median and interquartile ranges are shown unless indicated otherwise. A comprehensive table including all patients’ demographic and clinical characteristics can be found in Supplementary Table 1

	AIH	HC	PBC	PSC	NAFLD	Viral and alcoholic hepatitis		
						WC	CC	DC
**Patients**	66	60	10	31	30	15	29	30
**Number of samples [mean per patient]**	214 [3.2]	60 [1]	10 [1]	31 [1]	30 [1]	15 [1]	29 [1]	30 [1]
**Female gender [%]**	47 [71]	43 [72]	9 [90]	10 [32]	17 [57]	6 [40]	5 [17]	9 [30]
**Age female**	50 [27–62]	49 [32–63]	52 [50–53]	48 [36–66]	60 [49–67]	55 [51–55]	47 [45–57]	59 [57–63]
**Age male**	56 [32–63]	55 [32–63]	57 [-]	50 [38–61]	43 [29–62]	59 [48–61]	53 [50–57]	56 [47–59]
**Cirrhosis at first sample [%]**								
No cirrhosis	39 [59]		7 [70]	19 [61]	26 [87]	15 [100]	0 [0]	0 [0]
Compensated cirrhosis	22 [33]		3 [30]	11 [35]	3 [10]	0 [0]	29 [100]	0 [0]
Decompensated cirrhosis	5 [7.6]		0 [0]	1 [3]	1 [3]	0 [0]	0 [0]	30 [100]
**Laboratory values**								
AST (IU/L)	37 [26–59]		50 [35–54]	50 [38–92]	29 [24–42]	30 [17–37]	87 [45–116]	61 [49–99]
ALT (IU/L)	38 [23–67]		38 [28–77]	54 [37–106]	41 [36–71]	25 [21–57]	47 [26–70]	30 [25–40]
ALP (IU/L)	88 [64–120]		277 [158–433]	214 [148–319]	79 [70–95]	107 [94–125]	123 [95–145]	117 [104–166]
GGT (IU/L)	58 [25–163]		147 [58–571]	162 [86–256]	44 [29–60]	27 [21–69]	60 [38–93]	66 [30–96]
IgG (g/L)	12 [10–17]							
**Treatment response at first sample [at all timepoints]**								
No treatment	14 [19]							
No complete biochemical response	31 [115]							
Complete biochemical response	21 [75]							
**Medication at first timepoint [at all timepoints]**								
No medication	14 [19]							
Steroid based treatment	43 [136]							
Steroid free treatment	9 [58]							

AIH: autoimmune hepatitis; NAFLD: non-alcoholic fatty liver disease; PBC: primary biliary cholangitis; PBC: primary sclerosing cholangitis; WC: without cirrhosis; CC: compensated cirrhosis; DC: decompensated cirrhosis; AST: aspartate aminotransferase; ALT: alanine aminotransferase; GGT: gamma-glutamyl transferase

Patients who were not treated at the moment of sampling were defined as “before treatment”. This could be at diagnosis or at a relapse after cessation of immunosuppressive medication. Patients with CBR, defined as normalization of AST, ALT and IgG [6] were classified as “remission”, while “no remission” included patients with an incomplete response, loss of remission or relapse during treatment. Incomplete response was defined as increases in AST, ALT and IgG without normalization. Loss of remission was defined as AST or ALT between 1 and 3 x upper limit of normal while relapse was defined as AST or ALT > 3 times the upper limit of normal. Distinction between incomplete response, loss of remission or relapse was determined based on AST, ALT and IgG levels at the moment of sample collection. Presence of cirrhosis was based on liver histology. If liver histology was not available, liver elastography or liver ultrasound were used. Decompensated cirrhosis was defined as presence of ascites, varices bleeding, hepatocellular carcinoma, hepatorenal or hepatopulmonary syndrome.

**Table 2:** Baseline characteristics of AIH patients as stratified per treatment category. Median and interquartile ranges are shown unless indicated otherwise

	No treatment	No complete biochemical response	Complete biochemical response
**Patients**	14	31	21
**Sex (F/M)**	7/7	23/8	17/4
**Age female**	59 [51–64]	49 [28–64]	43 [26–57]
**Age male**	51 [37–66]	57 [30–62]	56 [38–67]
**Cirrhosis**			
No cirrhosis	9	17	13
Compensated cirrhosis	4	11	7
Decompensated cirrhosis	1	3	1
**Laboratory values**			
AST (IU/L)	169 [108–415]	60 [50–94]	26 [24–30]
ALT (IU/L)	189 [105–304]	63 [39–127]	19 [15–27]
ALP (IU/L)	161 [106–191]	112 [77–162]	62 [49–73]
GGT (IU/L)	236 [118–394]	119 [44–258]	34 [26–72]
IgG (g/L)	19 [13–22]	16 [13–18]	11 [8–11]
**Medication**			
No medication	14	-	-
Steroid based treatment	-	27	16
Steroid free treatment	-	4	5

### Mass spectrometry glycomics and data processing

**Fig. 1:**
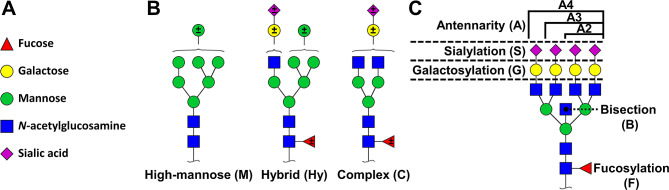
Monosaccharide diversity, glycan types and derived traits. (**A**) Common monosaccharide constituents of human *N*-glycans. (**B**) The three general types of *N*-glycans. (**C**) *N*-glycosylation derived traits, and their abbreviations, representing common biosynthetic pathways

The total plasma *N*-glycome of plasma samples was analyzed by matrix-assisted laser desorption ionization – Fourier-transform ion cyclotron resonance – mass spectrometry (MALDI-FTICR-MS) after sialic acid derivatization that allows to distinguish biologically important sialic acid linkage isomers, as described [29] (Supplementary Materials & Methods). IgG Fc glycosylation was analyzed at the glycopeptide level with nano-liquid chromatography coupled to MS (nLC-MS), allowing an IgG subclass and site-specific analysis as reported before [33]. After initial data pre-processing including data quality control, similarly to foregoing reports [17, 34] (Supplementary Materials & Methods), the relative abundances of individual glycans as well as of derived glycosylation traits (e.g. ratios of glycan abundances reflecting specific enzymatic biosynthetic steps) were calculated (Fig. 1, Supplementary Tables 2–4).

### Statistical analysis

A logistic regression model on standardized data (subtraction of the mean and division by the SD) including age, sex and their interaction as co-variates was used to study the associations between glycosylation of healthy controls (HC) and AIH patients (HC = 0; AIH = 1) (Table 3, Supplementary Table 5, Supplementary Materials & Methods). Glycosylation traits that were significantly different based on the logistic regression results were compared between disease groups using a Wilcoxon rank-sum test (Supplementary Tables 6–10, Supplementary Table 12, Supplementary Figs. 1–4), while a Wilcoxon signed-rank test was used to compare longitudinal timepoints corresponding to the same patient (Supplementary Table 11). To account for multiple testing, during the evaluation of statistical significance per statistical question (Table 3, Supplementary Tables 5–12, Benjamini-Hochberg procedure with a false discovery rate (FDR) of 5% was used.

**Table 3:** Associations between plasma and IgG *N*-glycan traits and AIH as compared to healthy controls**.** Logistic regression was performed between AIH (1) and HC (0), including age, sex and their interaction as co-variates. Only significant associations that passed the set log_2_ odds ratio threshold (Fig. 2A) are shown. The results of all tests can be found in Supplementary Table 5. To account for multiple testing, *p*-values have been corrected by the Benjamini-Hochberg procedure using a 5% FDR.

Derived traits	Description	*p*-value (age and sex corrected)	Odds ratio [95% CI] (age and sex corrected)	Beta coefficient	Standard error
**Glycan type**					
MHy	High mannose to hybrid-type ratio	1.62E-03	0.34 [0.2–0.56]	-1.06	0.27
THy	Total hybrid within total	2.31E-03	2.73 [1.67–4.89]	1.01	0.27
TA2FG0S0	Total fucosylated nongalactosylated nonsialylated A2	2.72E-03	18.55 [4.7-112.74]	2.92	0.81
TA2FS0	Total fucosylated nonsialylated A2	4.41E-03	3.3 [1.76–7.09]	1.19	0.35
**Bisection**					
CB	Of complex-type	5.88E-03	3.78 [1.88–9.35]	1.33	0.41
A2B	Of A2	6.10E-03	3.45 [1.79–7.99]	1.24	0.38
A2SB	Of sialylated A2	1.40E-02	2.76 [1.52–5.89]	1.01	0.34
A2FB	Of fucosylated A2	1.63E-03	3.21 [1.87–6.16]	1.17	0.30
A2FSB	Of fucosylated sialylated A2	2.72E-03	2.72 [1.65–4.96]	1.00	0.28
A2S0B	Of nonsialylated A2	2.23E-03	2.93 [1.74–5.39]	1.08	0.29
A2F0S0B	Of nonfucosylated nonsialylated A2	4.28E-03	2.57 [1.55–4.65]	0.94	0.28
A2F0B	Of nonfucosylated A2	2.12E-02	2.93 [1.49–6.89]	1.08	0.39
A2FS0B	Of nonfucosylated sialylated A2	2.72E-03	2.71 [1.64–4.86]	1.00	0.28
**Galactosylation**					
CG	Of complex-type	2.72E-03	0.06 [0.01–0.22]	-1.19	0.35
A2G	Of A2	2.77E-03	0.18 [0.06–0.42]	-1.70	0.48
A2FG	Of fucosylated A2	1.60E-03	0.28 [0.14–0.49]	-1.27	0.31
A2SG	Of sialylated A2	1.31E-02	0.33 [0.15–0.63]	-1.10	0.37
A2S0G	Of nonsialylated A2	1.60E-03	0.31 [0.17–0.52]	-1.17	0.29
A2FSG	Of fucosylated sialylated A2	2.72E-03	0.38 [0.21–0.62]	-0.97	0.27
A2FS0G	Of fucosylated nonsialylated A2	1.60E-03	0.32 [0.18–0.54]	-1.13	0.28
A2F0S0G	Of nonfucosylated nonsialylated A2	4.28E-03	0.35 [0.18–0.61]	-1.04	0.30
**Sialylation**					
CS	Of complex-type	4.28E-03	0.3 [0.14–0.57]	-1.19	0.35
A4S	Of A4	2.25E-03	2.7 [1.66–4.74]	0.99	0.27
A4GS	Per galactose on A4	1.63E-03	2.88 [1.75–5.16]	1.06	0.27
A4F0GS	Per galactose on nonfucosylated A4	2.72E-03	2.73 [1.64–4.92]	1.00	0.28
**IgG glycosylation derived traits**					
IgG1 bisection	IgG1 bisection	1.63E-03	2.55 [1.63–4.22]	0.94	0.24
IgG1 galactosylation	IgG1 galactosylation	3.47E-05	0.21 [0.11–0.37]	-1.55	0.30
IgG1 sialylation	IgG1 sialylation	1.52E-04	0.28 [0.16–0.45]	-1.29	0.27
IgG1 antennary fucosylation	IgG1 antennary fucosylation	1.62E-03	0.34 [0.19–0.56]	-1.07	0.27
IgG2/3 galactosylation	IgG2/3 galactosylation	1.62E-03	0.35 [0.2–0.58]	-1.04	0.26

CI: confidence interval

## Results

We studied a total of 66 patients with AIH. The majority of patients were female (71%) with a median age of 50 for females and 56 for males. 40.6% presented with cirrhosis at time of the first sample. Median ALT, AST and IgG were 37 IU/L, 38 IU/L and 12 g/L respectively (Tables 1 and 2, Supplementary Table 1). TPNG and IgG glycosylation were analyzed by MS, resulting in the relative quantification of 81 glycans for TPNG. For IgG1, IgG2/3 and IgG4 a total of 15, 12 and 13 glycans were quantified, respectively. The quantified glycoforms (Supplementary Tables 2–3) were summarized in derived glycosylation traits (Fig. 1, Supplementary Table 4). For the exclusively diantennary IgG glycans [35], these traits encompassed fucosylation, bisection, galactosylation and sialylation (Fig. 1, Supplementary Table 3). For the TPNG glycans traits, also antennarity and *N*-glycan type were included (Fig. 1, Supplementary Table 2). The identified glycoforms were characteristic for plasma proteins and IgG in agreement with literature [29, 35, 36].

### Plasma *N*-glycan and IgG-specific glycan traits associate with autoimmune hepatitis

**Fig. 2:**
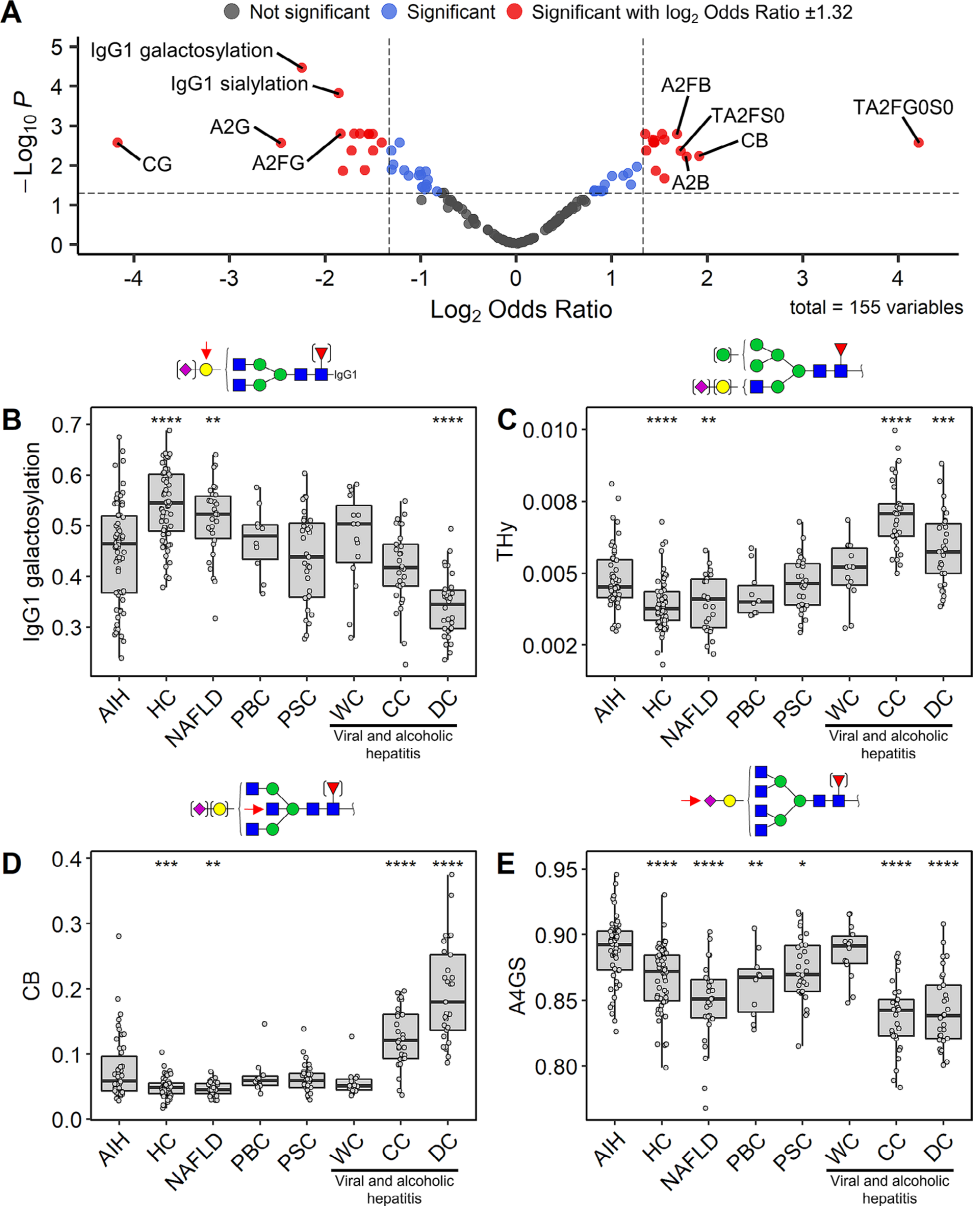
Main associations found between AIH patients and healthy controls. (**A**) Volcano plot based on the 155 derived glycosylation traits. (**B-E**) Relative abundance differences of derived glycosylation traits: (**B**) IgG1 galactosylation, (**C**) THy, (**D**) CB (**E**) A4GS, between AIH and healthy controls and other liver disease groups. AIH is the reference group against which comparisons are shown (Wilcoxon rank-sum test). *P*-values, ORs and corresponding 95% CIs for the associations are shown in Table 3 and Supplementary Table 5, whereas *p*-values for the disease group comparisons are shown in Supplementary Tables 6–7. Boxplots represent the median and the interquartile range, whilst whiskers correspond to the first and third quartiles (25th and 75th percentiles) and extend from the hinges to the largest or smallest value no further than 1.5x the interquartile range. *, **, ***, ****: *p*-value < 0.05, 0.01, 0.001, 0.0001, respectively

Comparing the derived glycan traits between AIH and healthy controls, 30 significant associations were found (Fig. 2A; Table 3). From these associations, the five glycosylation traits with the largest negative effect size (odds ratio (OR) between 0.28 and 0.06, corrected *p*-value (hereafter *p*-value) < 3 × 10^− 3^) were traits describing galactosylation levels in TPNG (A2G, CG, A2FG) as well as IgG1-specific galactosylation and sialylation (Fig. 2A; Table 3). The strongest positive effect size (OR between 3.21 and 18.55, *p*-value < 7 × 10^− 3^) was found for traits describing bisection levels in TPNG (A2B, CB, A2FB) as well as traits indicating the absence of galactoses and sialic acids on diantennary TPNG glycans (TA2FS0 and TA2FG0S0) (Fig. 2A; Table 3). All 30 glycosylation traits showing a difference between AIH and healthy controls were then further investigated in patients suffering from NAFLD, PBC, PSC and viral or alcoholic hepatitis (Supplementary Tables 6–7).

### TPNG Sialylation per galactose on tetraantennary glycans

Interestingly, we observed a higher sialylation per galactose on tetraantennary glycans (A4GS) as an AIH-specific plasma *N*-glycan signature (OR: 2.88, 95% confidence interval (CI) [1.75–5.16], *p*-value: 1.63 × 10^− 3^). A4GS was not only different in AIH patients when compared to HC, but also compared to all other disease groups in the cohort, except for patients with viral hepatitis without cirrhosis (WC) (Fig. 2E, Supplementary Tables 6–7). Importantly, A4GS is neither confounded by the age and sex of the patient, nor by cirrhosis occurrence or severity (Supplementary Figs. 3–4, Supplementary Table 9).

### TPNG Bisection

**Fig. 3:**
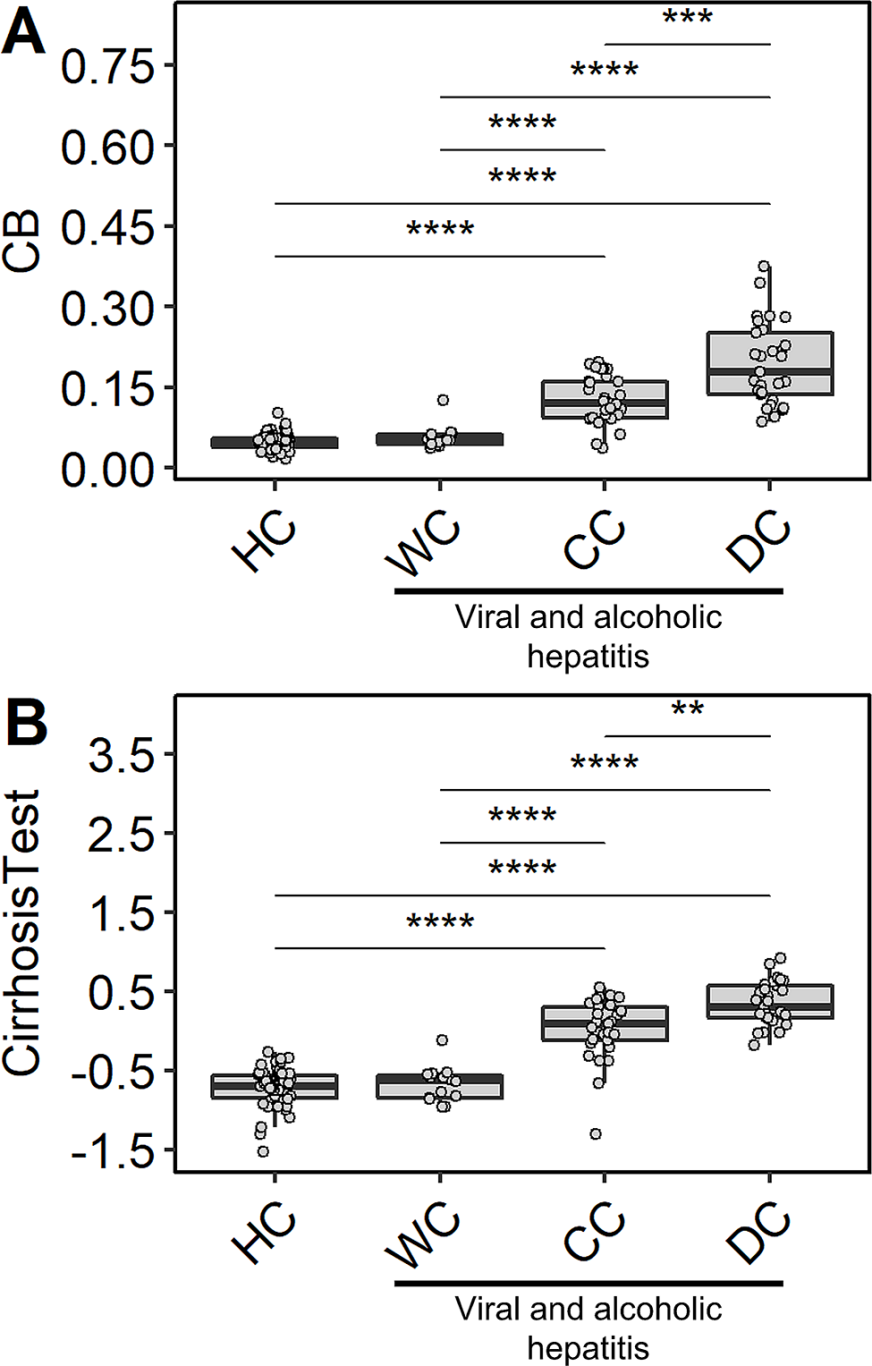
Associations between cirrhosis severity and bisection in alcoholic and viral hepatitis patients. Relative abundance differences of glycosylation traits (**A**) CB and (**B**) CirrhosisTest. Statistical tests and boxplots as described in Fig. 2

TPNG bisection (CB) associated positively with AIH as compared to HC as well as to NAFLD patients. As bisection is a known marker of cirrhosis [10, 11], the degree of TPNG bisection was monitored in alcoholic and viral hepatitis patients, who were stratified based on cirrhosis severity (Fig. 3; Table 1). CB showed no difference between healthy individuals and viral hepatitis patients without cirrhosis (Fig. 3A, Supplementary Table 8). Patients with compensated or decompensated cirrhosis (CC and DC, respectively) featured increased levels of bisection (CC fold change (FC): 2.47, *p*-value: 6.90 × 10^− 11^; DC FC: 3.67, *p*-value: 2.60 × 10^− 13^), with a more pronounced effect for DC (Fig. 3A, Supplementary Table 8). Similar observations were made when we used our data to simulate the GlycoCirrhoTest [10, 11] which measures the bisection of diantennary glycans, relatively to triantennary glycans (Fig. 3B, Supplementary Table 8). The reported cirrhosis effect was also seen within the AIH patients (Supplementary Fig. 4, Supplementary Table 9).

### IgG galactosylation

Although IgG1 galactosylation was decreased in AIH patients as compared to HC, this was not unique for AIH but occurred in most of the disease groups, except NAFLD (Fig. 2B, Supplementary Tables 5–6). Patients suffering from hepatitis with DC featured an even lower IgG galactosylation than the AIH patients (Fig. 2B, Supplementary Tables 6–7). A negative relation between IgG1 galactosylation and cirrhosis severity was observed for the hepatitis as well as the AIH patients (Fig. 2B, Supplementary Figs. 2, 4; Supplementary Tables 8–9).

### TPNG hybrid-type glycans

The total level of hybrid-type glycans (THy) in AIH was higher as compared to HC and NAFLD patients but had no discriminative power towards the other liver diseases. THy showed a positive association with the occurrence of cirrhosis, both for the hepatitis and the AIH patients (Fig. 2C; Table 3, Supplementary Tables 6–7).

### Plasma *N*-glycan and IgG-specific glycan traits associate with treatment and disease activity in AIH

**Fig. 4:**
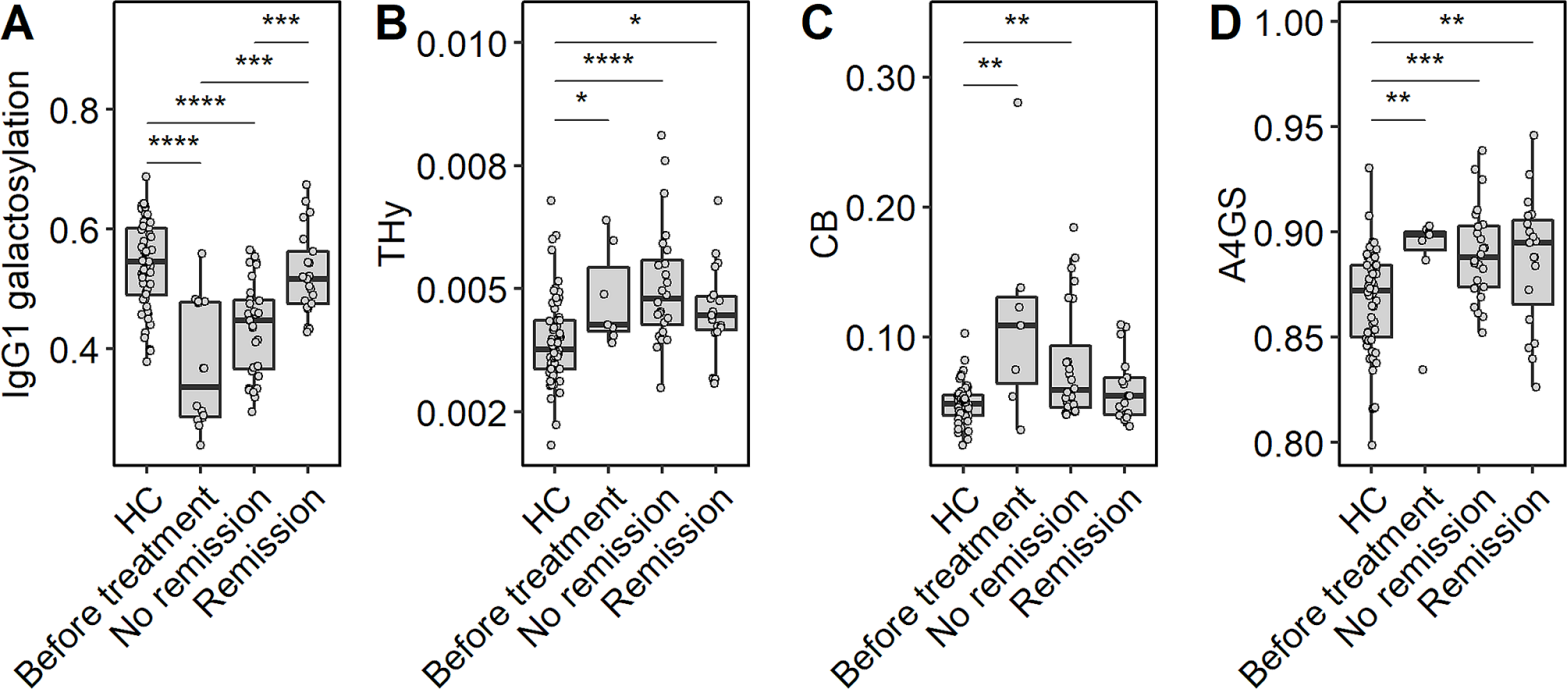
Correlations between AIH disease activity and glycosylation. Relative abundance differences of derived glycosylation traits: (**A**) IgG1 galactosylation, (**B**) THy, (**C**) CB and (**D**) A4GS. Statistical tests and boxplots as described in Fig. 2

To investigate glycosylation features associated with disease activity, we separated the first available sample per patient based on treatment response (i.e., before treatment, no remission or CBR) (Table 2). IgG1 galactosylation of patients with active disease (i.e., before treatment or not in remission) was lower as compared to HC (FC: 0.6158, *p*-value: 4.2 × 10^− 6^), while patients with CBR did not show an IgG galactosylation effect as compared to HC (Fig. 4A, Supplementary Table 10). The same was observed for CB, which was significantly different between patients with an active disease as compared to HC, while no difference was seen for patients with CBR. However, when following these traits longitudinally in patients developing from CBR to no remission or vice versa, no differences in IgG galactosylation or CB were observed. Within the treated patients, a slightly higher level of IgG galactosylation was found for the steroid treated group, as compared to non-treated, while other comparisons did not reveal any differences (Supplementary Table 12).

**Fig. 5:**
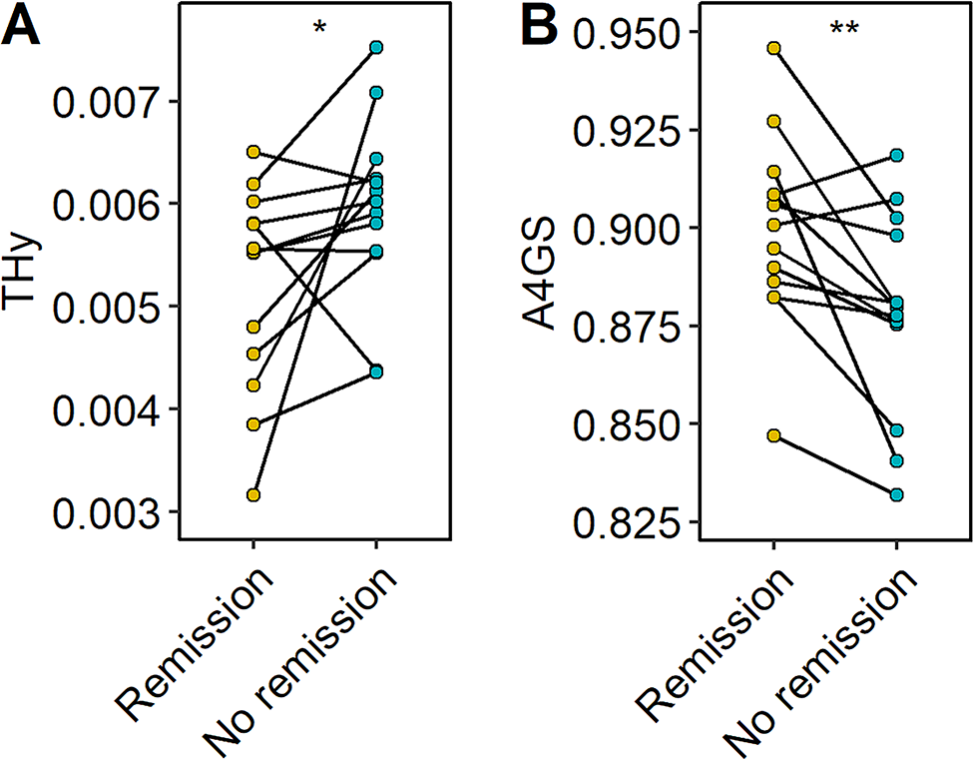
Longitudinal changes associated with AIH disease activity. Relative abundance differences of glycosylation derived traits: (**A**) THy and (**B**) A4GS. A Wilcoxon signed-rank test was used to compare longitudinal samples (*n* = 11)

The traits THy and A4GS were consistently higher in AIH patients as compared to healthy controls, independent of their treatment status (Fig. 4B, D; Supplementary Table 10). Interestingly, a change was observed for both glyco-traits in a longitudinal manner in patients developing from CBR to no remission, with THy increasing, and A4GS decreasing (Fig. 5, Supplementary Table 11). The reverse effect (i.e., for patients developing from no remission to CBR) was not observed (Supplementary Fig. 5, Supplementary Table 11).

## Discussion

In this cross-sectional cohort study, elevated A4GS was found as a unique marker in patients with AIH compared to healthy controls and other liver diseases. Liver inflammation and cirrhosis were shown to be important confounders influencing glycosylation patterns, but A4GS was increased in AIH patients independently of these factors. By our knowledge, this is the first study to extensively investigate glycosylation in AIH compared to healthy controls and other liver diseases and the findings offer opportunities to facilitate the non-invasive and accurate diagnosis of AIH.

Currently, diagnosis of AIH is based on the revised [2] or the simplified [3] AIH criteria. Although these clinical criteria exist, in practice it can be challenging to distinguish AIH from PBC, PSC and NAFLD, and a liver biopsy is in any case required for diagnosis. To reduce the invasive and risky need for liver biopsies, blood-derived markers to distinguish the diseases are desirable. We used an exploratory, MALDI-FTICR-MS-based approach for studying plasma protein *N*-glycosylation, with high glycoform resolution and the ability to study sialylation and its linkages [29]. Our approach identified high A4GS as an AIH-specific plasma *N*-glycan signature, which was not only higher in AIH patients as compared to HC, but also as compared to most other liver disease groups in the cohort. This marked elevation effectively distinguished AIH patients from HC irrespective of remission status. Only patients with viral or alcoholic hepatitis without cirrhosis featured high levels of A4GS as well, but in the clinic the differentiation between viral or alcoholic hepatitis and AIH is straightforward using conventional diagnostics. The observed effect was specific for tetraantennary glycans, and independent of sialic acid linkage (Supplementary Table 5). Of note, the sialylation per galactose of di- (A2GS) and triantennary glycans (A3GS) did not show the above described trend (Supplementary Table 5), indicating it is not a global sialylation effect. Furthermore, no significant differences or associations between IgG, ALT or AST levels at diagnosis and A4GS levels were found. Despite the limited number of patients, this suggests that the high A4GS signature at diagnosis may be associated with AIH rather than the level of disease activity and may therefore be helpful in the diagnostic workup. This should be further investigated in larger studies.

Sialylated tetraantennary glycans in human plasma largely originate from alpha-1-acid glycoprotein (AGP) [35]. AGP is a major positive acute phase protein, that functions both as an immunomodulatory as well as a transport protein, harbours 5 *N*-glycosylation sites and is characterized by remarkable glycosylation microheterogeneity [37–39]. Hitherto, reports on AGP glycosylation mainly highlighted alterations in branching and (antennary) fucosylation to be associated with (liver) diseases [40, 41], and more recently sialylation in NASH and HCC [12]. The increased level of sialylation on tetraantennary glycans found in the plasma of AIH patients is a novel finding, which can either be an effect of altered regulation of sialylation or a proxy showing the upregulation of highly sialylated glycoproteins, such as AGP. Factors that regulate the levels of sialylation of circulatory proteins are, amongst others, the abundance and activity of sialyltransferases in the glycoprotein producing cells, the availability of CMP-sialic acid and the removal of non-sialylated proteins from the circulation by the asialoglycoprotein receptor (ASGPR) in the liver. While investigating the expression levels of sialyltransferases in liver cells associated with AIH would be a fruitful direction for further research, a role of the ASGPR in the observed effects seems unlikely. AIH is associated with an upregulation of ASGPR-specific autoantibodies [42], which would have an opposite effect on the level of sialylation in the circulation, as the asialylated proteins will be limitedly removed.

While the current findings are a promising starting point in the development of a non-invasive diagnostic strategy for AIH, replication studies are required that confirm the observed effect. In addition, the marker has the potential to increase in specificity when the plasma/liver glycoproteins are identified that are responsible for the observed differences. An obvious glycoprotein candidate to further investigate is AGP and efforts to study levels and glycosylation of AGP in AIH patients are highly recommended. To consolidate our findings on the longitudinal intra-patient variation observed for A4GS and its potential to monitor disease activity over time, future prospective studies should involve longitudinal samples from treatment naïve patients with varying clinical presentations. Despite the long-term follow-up in the current study, samples were intermittently distributed in time and showed a large heterogeneity in inflammation, cirrhosis, and treatment status as well as treatment type. This, in combination with limited sample numbers, may have caused an inherent bias when longitudinal changes in altering inflammation categories were compared. Additionally, it is important to evaluate the discriminative function of the glycosylation signature in a control cohort of patients with drug-induced liver injury (DILI), since AIH and DILI are sometimes difficult to discern in clinical practice.

Other differentiators between AIH patients and HC were TPNG- and IgG bisection. The degree of cirrhosis in liver diseases vastly confounded the bisection effect in our study and this trait appeared rather cirrhosis-specific than AIH-specific, as described in literature, and exploited in the clinically approved GlycoCirrhoTest [10, 11]. The bisection effect is likely derived from IgG, as supported by the IgG-specific data, but IgM and IgA, that are also known to carry diantennary glycans with bisecting *N*-acetylglucosamines, may contribute to this observation [35].

The level of hybrid type glycans in plasma associated with the occurrence of cirrhosis and showed a trend towards increased levels with flaring AIH. However, for the evaluation of THy as marker for disease activity, replication is needed in a more uniform set of longitudinal follow-up samples, accounting for cirrhosis and the use of medication. Higher levels of hybrid-type structures are worth further investigation in the context of AIH, as hybrid-type glycans on vitronectin have been reported to be elevated in HCC patients [43, 44]. Conversely, plasma-derived hybrid-type glycans negatively associated with Crohn’s disease and ulcerative colitis in a cross-sectional study [16].

Increased plasma IgG levels are one of the hallmarks of AIH, which motivated the independent investigation of IgG-specific glycosylation. Part of the galactosylation effects observed in plasma (a decreased A2G, CG, A2FG in most liver diseases) were explained by IgG-specific changes [35]. The undergalactosylation of IgG is a known marker of ongoing inflammation, which is well documented in a broad range of diseases such as RA [45, 46] and other autoimmune diseases [47–49], IBD [16, 22], colorectal cancer [50], infectious diseases [51, 52], as well as upon aging [24–26, 53]. The underlying biological mechanism that might be responsible for the pro-inflammatory nature of undergalactosylated IgG is its potential ability to elicit complement activation via binding to the mannose-binding lectin [21]. However, this causal relationship was not proven and agalactosylated total plasma IgG can also be a mere effect of the inflammatory process. In addition, complement activation does not appear to play a major role in AIH [54]. There is a large body of evidence correlating IgG galactosylation with inflammatory markers and disease activity [53, 55, 56], and lower IgG1 galactosylation correlated to disease activity in the current cross-sectional samples. However, no galactosylation effect was observed in the longitudinal sampling of patients changing their inflammatory status, which suggests that AIH patients in this cohort show too much heterogeneity in disease activity, cirrhosis severity and use of medication to use IgG galactosylation as marker for inflammation.

Our study focused on total plasma *N*-glycosylation and IgG glycosylation in an integrated fashion, which is unique in the context of high-throughput glycomics. Still, further studies are needed to confirm the source protein from which the observed A4GS effect derives from. The currently used glycoanalytical methodologies are not yet suitable for direct clinical application. As the clinically approved GlycoCirrhoTest unfortunately does not feature sialylation analysis [10, 11], suitable alternatives should be developed to further implement glycosylation analysis in clinical practice. These may be based on (immune)affinity assays targeting sialylation, or exploit techniques that provide a higher glycoform resolution, such as capillary electrophoresis [57].

In conclusion, by characterizing total plasma *N*-glycosylation and IgG glycosylation, an AIH-specific glycosylation profile was found. High A4GS is unique for AIH and independent of remission status, which offers possibilities for development of new diagnostic markers, potentially reducing the need for liver biopsy. Secondly, it can also offer a new perspective on pathophysiology of AIH. Glycosidic changes related to disease activity should be investigated further and might aid physicians with monitoring of disease activity in the future.

### Electronic supplementary material

Below is the link to the electronic supplementary material.

Supplementary Material 1

Supplementary Material 2

## Data Availability

Raw mass spectrometry data is available from the corresponding author upon reasonable request.

## Abbreviations

AGP: Alpha–1–acid glycoprotein
AIH: Autoimmune Hepatitis
ALT: Alanine Transaminase
AST: Aspartate Transaminase
ASGPR: Asialoglycoprotein Receptor
A4GS: Tetraantennary Sialylation Per Galactose
CBR: Complete Biochemical Response
CC: Compensated Cirrhosis
CI: Confidence Interval
COVID-19: Coronavirus Disease 2019
DC: Decompensated Cirrhosis
FC: Fold Change
Fc: Fragment crystallizable region
FDR: False Discovery Rate
FTICR: Fourier Transform Ion Cyclotron Resonance
HC: Healthy Controls
HCC: Hepatocellular Carcinoma
HCV: Hepatitis C virus
IBD: Inflammatory Bowel Disease
IgG: Immunoglobulin Gamma
LC: Liquid Chromatography
MALDI: Matrix-Assisted Laser Desorption/Ionization
MS: Mass Spectrometry
NAFLD: Non–Alcoholic Fatty Liver Disease
NASH: Non–Alcoholic Steatohepatitis
OR: Odds Ratio
PBC: Primary Biliary Cholangitis
PSC: Primary Sclerosing Cholangitis
RA: Rheumatoid Arthritis
TPNG: Total Plasma *N*–Glycosylation
WC: Without Cirrhosis
