# Supplementary material for: Autoimmune hepatitis displays distinctively high multi-antennary sialylation on plasma N-glycans compared to other liver diseases
Source: J Transl Med. 2024 May 14;22:456. doi: 10.1186/s12967-024-05173-z (PMC11092172; doi:10.1186/s12967-024-05173-z)
Supplement: Supplementary file 2 — Supplementary Material 2 [file 12967_2024_5173_MOESM2_ESM.docx]

**Autoimmune hepatitis displays a unique plasma *N*-glycan signature as compared to other liver diseases**

Tamas Pongracz^1,#^, Maaike Biewenga^2,#^, Anna Eva Charlotte Stoelinga^2^, Marco René Bladergroen^1^, Simone Nicolardi^1^, Leendert Adrianus Trouw^3^, Manfred Wuhrer^1^, Noortje de Haan^1,*^, Bart van Hoek^2,*^

^1^Center for Proteomics and Metabolomics, Leiden University Medical Center, Albinusdreef 2, 2333 ZA Leiden, The Netherlands

^2^Department of Gastroenterology and Hepatology, Leiden University Medical Center, Albinusdreef 2, 2333 ZA Leiden, The Netherlands

^3^Department Immunohematology and Blood Transfusion, Leiden University Medical Center, Albinusdreef 2, 2333 ZA Leiden, The Netherlands

^#^ These authors share co-first authorship; ^*^ These authors share co-senior authorship

Corresponding author: Noortje de Haan, [n.de_haan@lumc.nl](mailto:n.de_haan@lumc.nl)

**Table of contents**

[Supplementary Materials & methods 3](#_Toc79669822)

[Materials 3](#_Toc79669823)

[Sample preparation for high-throughput glycosylation analysis workflows 3](#_Toc79669824)

[Glycan release from plasma, linkage specific sialic acid stabilization and MALDI-FTICR-MS analysis 3](#_Toc79669825)

[Isolation of IgG from plasma/serum, glycopeptide generation and nanoLC-MS analysis 4](#_Toc79669826)

[Data processing 4](#_Toc79669827)

[Derived trait calculation 5](#_Toc79669828)

[Calculation of the CirrhosisTest 5](#_Toc79669829)

[Method repeatability and robustness 5](#_Toc79669830)

[Statistics 5](#_Toc79669831)

[Supplementary Figures 6](#_Toc79669832)

[References 12](#_Toc79669833)

# **Supplementary Materials & methods**

## *Materials*

Materials and reagents used in the study were of analytical grade and purchased from commercial suppliers. Type I Ultrapure Water (UP) was used to prepare solutions, which was produced by an ELGA Purelab Ultra system (Elga LabWater, High Wycombe, United Kingdom). Nonidet P-40 substitute (NP-40), super-DHB and 1-hydroxybenzotriazole monohydrate (HOBt), ammonium bicarbonate (NH_4_HCO_3_), potassium chloride (KCl), disodium hydrogen phosphate hydrate (Na_2_HPO_4_∙7H_2_O) formic acid, 85% phosphoric acid (H_3_PO_4_) and tolylsulfonyl phenylalanyl chloromethyl ketone (TPCK)−treated trypsin from bovine pancreas were obtained from Sigma-Aldrich (Steinheim, Germany). Ethanol, sodium hydroxide (NaOH), sodium dodecyl sulphate (SDS), and trifluoroacetic acid, disodium hydrogen phosphate dihydrate (Na_2_HPO_4_∙2H_2_O), potassium dihydrogen phosphate (KH_2_PO_4_), and sodium chloride (NaCl) were purchased from Merck (Darmstadt, Germany). 1-ethyl-3-(3-(dimethylamino)propyl)carbodiimide hydrochloride (EDC) was obtained from Fluorochem (Hadfield, United Kingdom), while peptide-*N*-glycosidase F (PNGase F) was purchased from Roche Diagnostics (Mannheim, Germany). HPLC-supra-gradient acetonitrile (ACN) and ethanol (EtOH) were obtained from Biosolve (Valkenswaard, The Netherlands) and Merck (Darmstadt, Germany), respectively. The Visucon-F healthy human plasma standard originated from Affinity Biologicals (Ancaster, Canada). Peptide Calibration Mix II was made available by Bruker Daltonics (Bremen, Germany). Protein G Sepharose 4 Fast Flow beads were obtained from GE Healthcare (Uppsala, Sweden).

## *Sample preparation for high-throughput glycosylation analysis workflows*

Samples **(Supplementary Table 1)** were randomized on 5 96-well plates, together with 4 Visucon F standards, 3 pooled serum standards (created by pooling equal amounts of sera originating from AIH patients) and 2 or 3 blanks per plate. Age and sex were taken into account for an optimal distribution of cases, controls, and disease groups per plate. Randomization minimizes biases, ensuring even variable distribution. Sex- and age-matching controls and disease group distribution enhance comparability and representative sampling, promoting statistical validity in high-throughput experiments.

## *Glycan release from plasma, linkage specific sialic acid stabilization and MALDI-FTICR-MS analysis*

Release of *N*-glycans from plasma proteins and linkage-specific chemical sialic acid derivatization was performed as previously described in similar high-throughput, robotized workflow, using 2 uL of plasma/serum for the release^1-3^. For MALDI-FTICR-MS measurement, 1 uL sDHB matrix was topped by 1uL HILIC-purified sample and left to dry by air^1-3^. The measurement was performed on a 15 T Bruker SolariX XR FTICR mass spectrometer equipped with a ParaCell, a Smartbeam-II laser and a Combisource (Bruker Daltonics, Bremen, Germany) in positive ionization mode^2,3^. Calibration was performed with Peptide Calibration Mix II (Bruker Daltonics).

## *Isolation of IgG from plasma/serum, glycopeptide generation and nanoLC-MS analysis*

IgG was isolated from plasma or serum using protein G affinity chromatography, as described previously^4^. After tryptic digestion of the IgG, an Ultimate 3000 high-performance liquid chromatography (HPLC) system (Dionex Corporation, Sunnyvale, CA) was used for the separation of glycopeptides, which was hyphenated to a Maxis Impact HD quadrupole time-of-flight mass spectrometer (Bruker Daltonics) used for the detection the different Fc-glycans, as described^4^. As tryptic IgG2 and IgG3 glycopeptides were assumed to share a common peptide backbone, they were indistinguishable with our methods^5^.

## *Data processing*

MALDI-FTICR-MS raw spectra were converted into xy files, whereas mzXML files were generated from the nanoLC-MS raw spectra. Extraction of these raw data was performed using in-house developed software MassyTools^6^ and LaCyTools^7^, respectively. Chromatograms were aligned based on average retention time and exact mass of five highest abundant glycoforms in each IgG cluster. For the targeted extraction of glycan and glycopeptide peaks, analyte lists were created based on manual annotation of summed mass spectra per disease group. The assignment of glycoforms was based on exact mass and previous reports^2,8-10^. For MALDI-FTICR-MS data, the 1+ charge state was used for extraction, whilst the 2+ and 3+ charge states were used for nanoLC-MS data extraction. Signals were integrated by covering minimum 95% of the area of the isotopic envelope of glycan and glycopeptide peaks. Serum samples (considering 26 AIH and 2 PSC patients) **(Supplementary Table 1)** were excluded from the TPNG analysis, because serum, unlike plasma, is depleted from blood clotting factors, such as the abundant glycoprotein fibrinogen, making comparison of the two inherently biased. An analyte was included in the final data analysis if its signal-to-noise was above 27, its isotopic pattern did not deviate more than 25% from the theoretical one, and if its mass error was within a ±20 parts per million range. Additionally, the same analyte (glycan or glycopeptide for TPNG and IgG, respectively) had to be present in at least 1 out of 4 spectra (25%) in each disease group for inclusion to the final data analysis. The relative intensity values of glycan compositions that passed quality criteria were calculated by normalizing to the sum of their total areas in case of TPNG, and per subclass for IgG.

## *Derived trait calculation*

Based on the measured direct plasma derived glycan traits (n = 81) and glycopeptide traits (IgG1, IgG2/3 and IgG4; n = 15, 12, 13, respectively), derived glycosylation traits were calculated based on common structural characteristics, including the number of antennae (CA), and the levels of fucosylation (F), bisection (B), galactosylation (G), or sialylation (S), **(Fig. 1, Supplementary Table 4)**.

## *Calculation of the CirrhosisTest*

GlycoCirrhoTest is a clinically used test and is calculated by the logarithmic transformation of the absolute abundance ratio of diantennary fucosylated glycans containing a bisected *N*-acetlyglucosamine (H5N5F1) to tri-antennary non-fucosylated (H6N5) glycans after enzymatic desialylation^11,12^. CirrhosisTest is based on the calculation of the GlycoCirrhoTest^11,12^, yet includes sialylated analytes **(Supplementary Table 4)**.

## *Method repeatability and robustness*

To assess the MALDI-FTICR-MS method repeatability, the inter-plate coefficient of variation of the most abundant glycan peak H5N4E2 was calculated for the plasma standards and was 4.9%. To assess the nanoLC-MS method repeatability, the inter-plate variation of the most abundant glycan peak IgG1H4N4F1 was calculated for the plasma and serum standards and were 1.0% and 1.7%, respectively. The observed variations are in alignment with those commonly observed in our lab. To confirm that the observed effects were not batch associated, a principal component analysis was performed, displaying that the separation of standards was not driven by systematic batch effects **(Supplementary Fig 6)**.

## *Statistics*

Age, sex and their interaction were included as co-variates in a logistic regression model to find disease specific associations of HC (0) versus AIH (1). The odds ratios (OR) were calculated with their 95% confidence intervals (CI) and represent single standard deviation increases in the tested derived traits. Multiple testing correction was performed using the Benjamini-Hochberg procedure and was based on a false discovery rate (FDR) of 5%. The statistical analyses were performed in R, version 4.0.3 (R Foundation for Statistical Computing, Vienna, Austria) and RStudio, version 1.4.1103 (RStudio, Boston, MA).

# **Supplementary Figures**

**
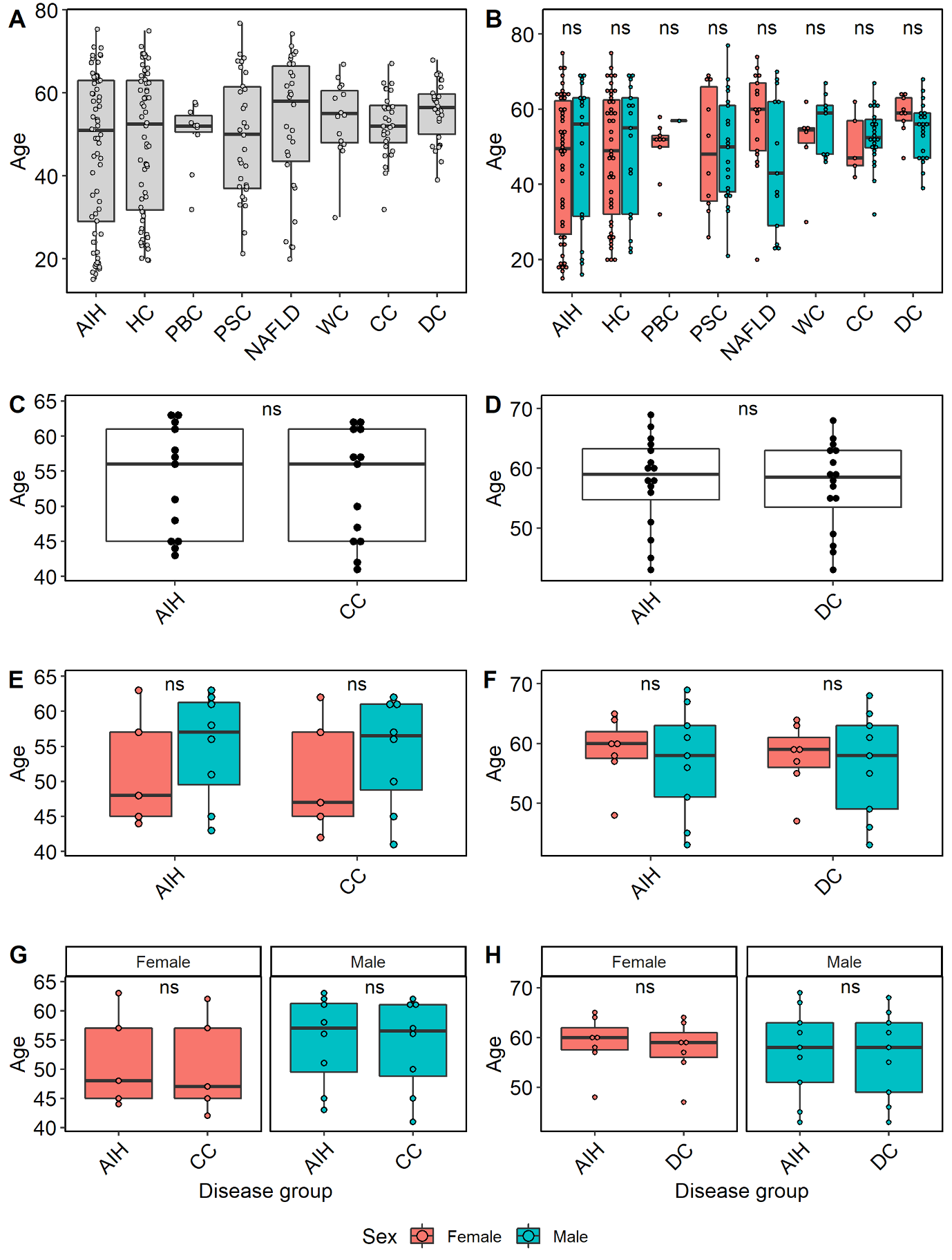
**

**Fig. S1. Age and sex distribution across disease groups, and in an age and sex matched subset of AIH, CC and DC. (A)** Age distribution in the cohort. **(B)** Age distribution across disease groups, as stratified per sex. (**C-H)** Age and sex distribution in an age and sex matched subset of AIH, CC and DC, for the visualization of potential confounding effect of age and sex. No significant age and sex differences were observed. ns: no significance. Statistical tests and boxplots as described in **Fig 2.**

**
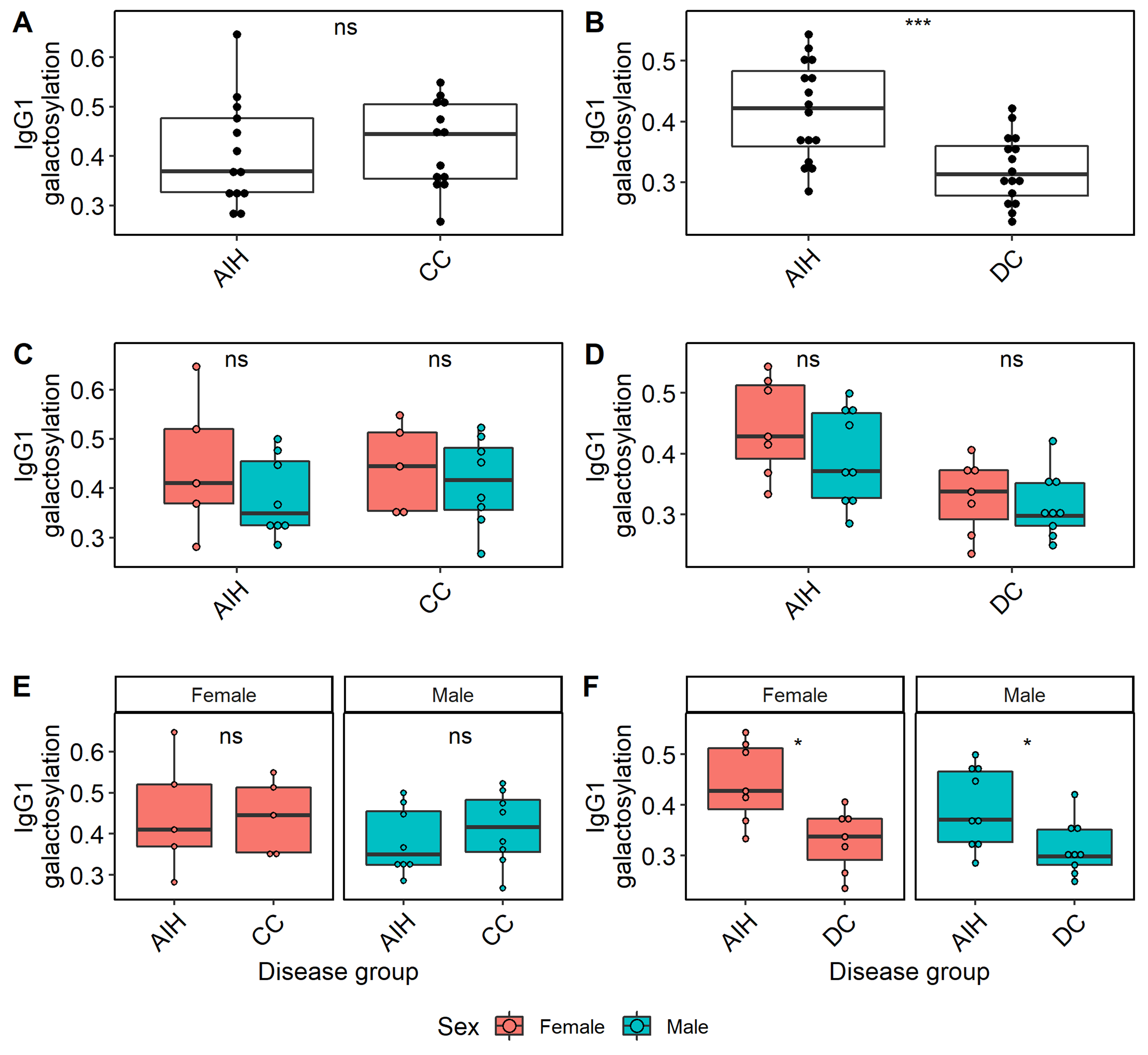
**

**Fig. S2.** IgG1 galactosylation as shown for an age and sex matched subset of AIH, CC and DC, for the visualization of potential confounding effect on age and sex, when compared to effects seen on **Fig. 2B**. A disease effect could be confirmed for the hepatitis patients with decompensated cirrhosis **(D, F)**, unlike for hepatitis patients with compensated cirrhosis, for which a potential age-effect could not be excluded **(C, E)**. Statistical tests and boxplots as described in **Fig 2.**


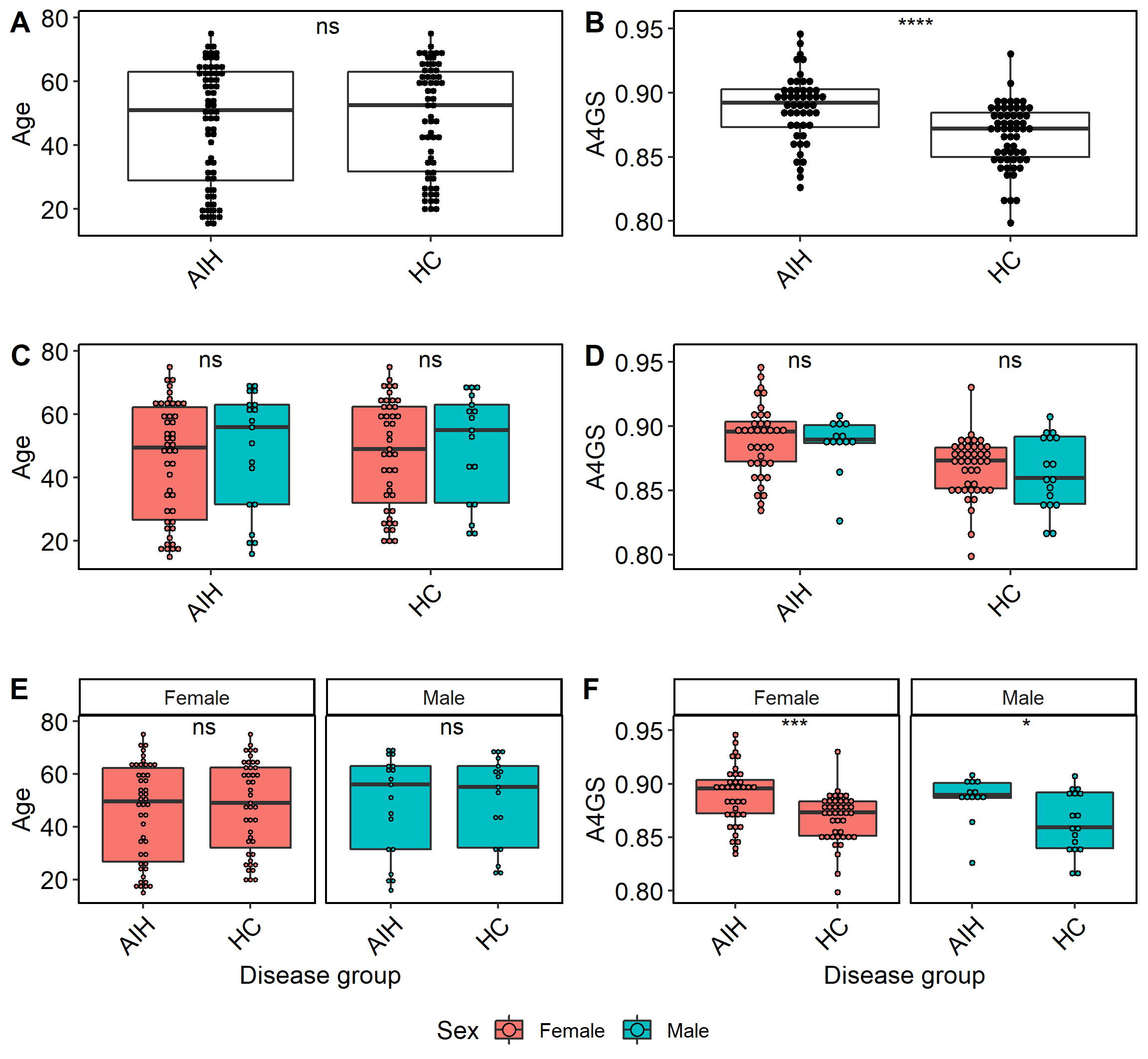


**Fig. S3.** A4GS as shown for the age and sex matched AIH patients and HC, for the visualization of potential confounding effect on age and sex. A disease effect could be confirmed for AIH. Statistical tests and boxplots as described in **Fig 2.**


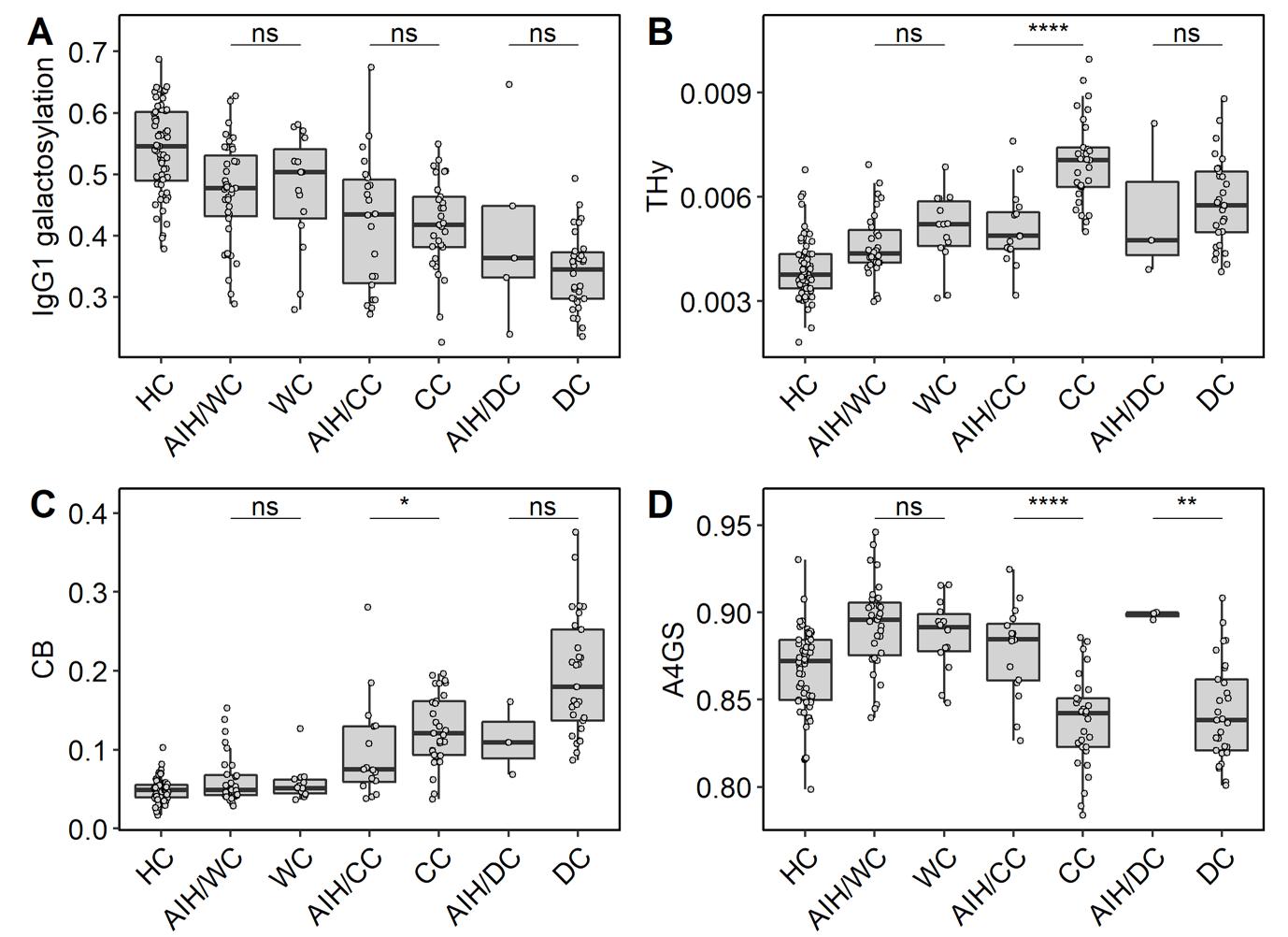


**Fig. S4.** **Comparison of *N*-glycosylation signatures in patients with different degrees of cirrhosis.** Relative abundance differences of glycosylation derived traits **(A)** IgG1 galactosylation, **(B)** THy, **(C)** CB and **(D)** A4GS. HC: healthy controls; AIH/WC: autoimmune hepatitis without cirrhosis; AIH/CC: autoimmune hepatitis with compensated cirrhosis; AIH/DC: autoimmune hepatitis with decompensated cirrhosis. Patients without AIH in WC correspond only to viral hepatitis, whereas CC and DC are a mixture of alcoholic and viral hepatitis **(Table 1)**. Statistical tests and boxplots as described in **Fig 2**.


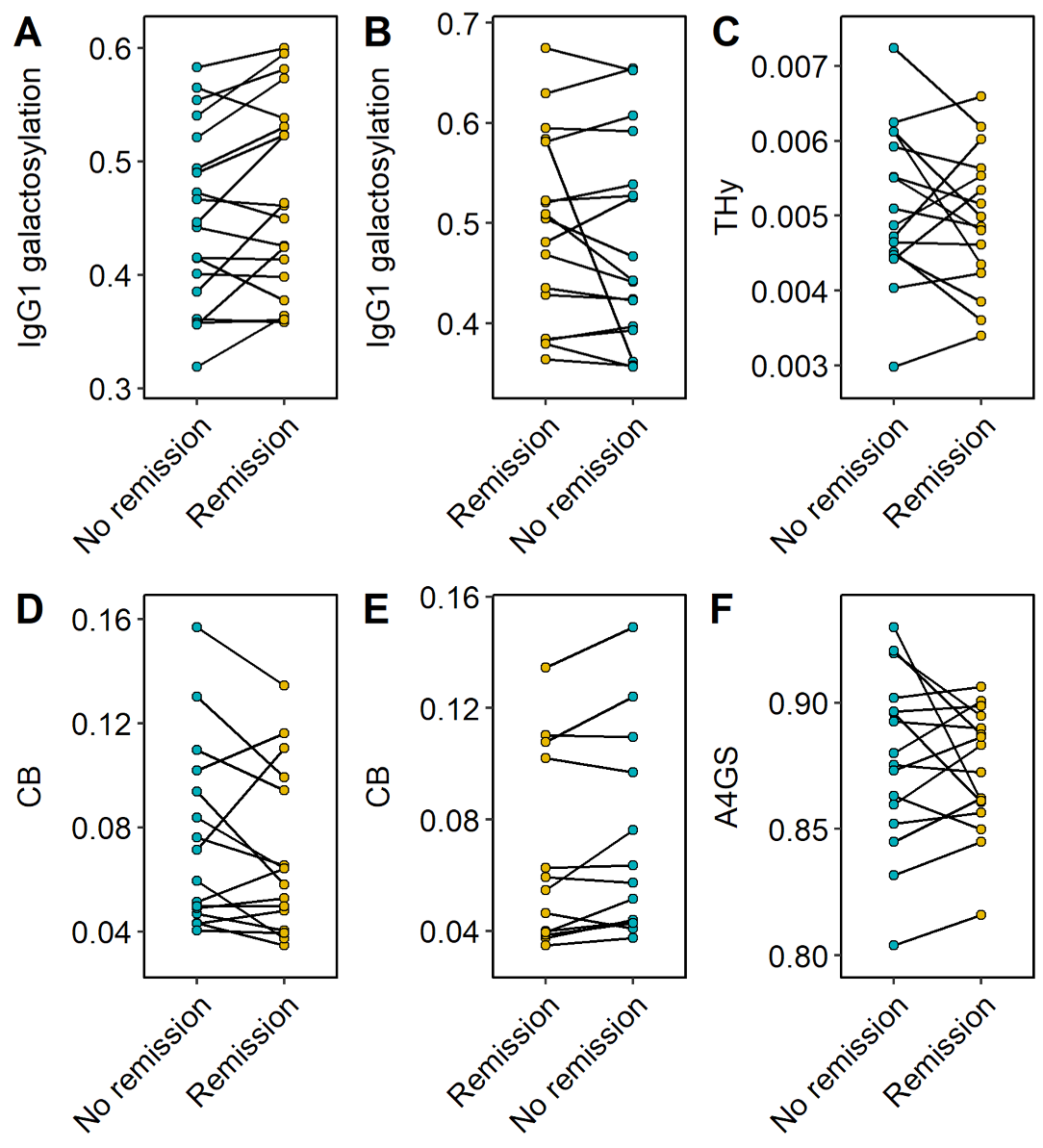


**Fig 5.** **Longitudinal changes showing no association with AIH disease activity.** Relative abundance differences of glycosylation derived traits: **(A-B)** IgG1 galactosylation, **(C)** THy, **(D-E)** CB and **(F)** A4GS.

**
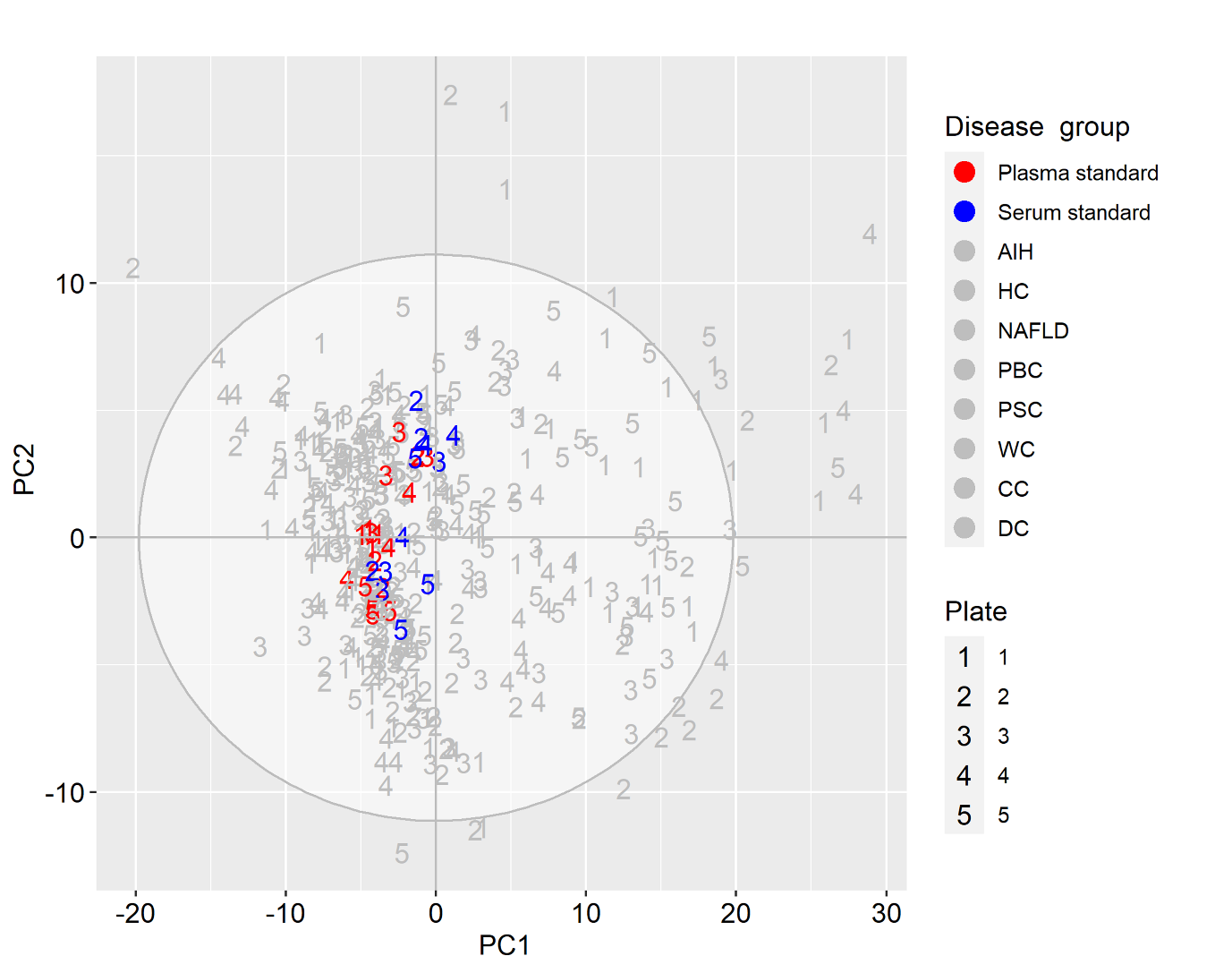
**

**Fig. S6. Principal component analysis (PCA) scores plot based on the distribution of sample plates per standards and disease groups.** Displayed is the 1^st^ PC against the 2^nd^ PC (covering 41.8% and 13.2% of the variation, respectively), with the ellipse indicating the 95% confidence interval. The separation of standards is not or hardly driven by systematic batch effects: the standards do not cluster per plate, indicating that the variation between standards is largely random and not batch-associated.

# **References**

1 Bladergroen, M. R. *et al.* Automation of High-Throughput Mass Spectrometry-Based Plasma N-Glycome Analysis with Linkage-Specific Sialic Acid Esterification. *Journal of Proteome Research* **14**, 4080-4086, doi:10.1021/acs.jproteome.5b00538 (2015).

2 Vreeker, G. C. M. *et al.* Automated Plasma Glycomics with Linkage-Specific Sialic Acid Esterification and Ultrahigh Resolution MS. *Anal Chem* **90**, 11955-11961, doi:10.1021/acs.analchem.8b02391 (2018).

3 Singh, S. S. *et al.* Metformin and statin use associate with plasma protein N-glycosylation in people with type 2 diabetes. *BMJ Open Diabetes Res Care* **8**, doi:10.1136/bmjdrc-2020-001230 (2020).

4 Falck, D., Jansen, B. C., de Haan, N. & Wuhrer, M. High-Throughput Analysis of IgG Fc Glycopeptides by LC-MS. *Methods Mol Biol* **1503**, 31-47, doi:10.1007/978-1-4939-6493-2_4 (2017).

5 Vidarsson, G., Dekkers, G. & Rispens, T. IgG subclasses and allotypes: from structure to effector functions. *Front Immunol* **5**, 520, doi:10.3389/fimmu.2014.00520 (2014).

6 Jansen, B. C. *et al.* MassyTools: A High-Throughput Targeted Data Processing Tool for Relative Quantitation and Quality Control Developed for Glycomic and Glycoproteomic MALDI-MS. *J Proteome Res* **14**, 5088-5098, doi:10.1021/acs.jproteome.5b00658 (2015).

7 Jansen, B. C. *et al.* LaCyTools: A Targeted Liquid Chromatography-Mass Spectrometry Data Processing Package for Relative Quantitation of Glycopeptides. *J Proteome Res* **15**, 2198-2210, doi:10.1021/acs.jproteome.6b00171 (2016).

8 Klein, A. Human total serum N-glycome. *Adv Clin Chem* **46**, 51-85, doi:doi.org/10.1016/S0065-2423(08)00402-2 (2008).

9 Clerc, F. *et al.* Human plasma protein N-glycosylation. *Glycoconj J* **33**, 309-343, doi:10.1007/s10719-015-9626-2 (2016).

10 Pucic, M. *et al.* High throughput isolation and glycosylation analysis of IgG-variability and heritability of the IgG glycome in three isolated human populations. *Mol Cell Proteomics* **10**, M111 010090, doi:10.1074/mcp.M111.010090 (2011).

11 Callewaert, N. *et al.* Noninvasive diagnosis of liver cirrhosis using DNA sequencer-based total serum protein glycomics. *Nat Med* **10**, 429-434, doi:10.1038/nm1006 (2004).

12 Verhelst, X. *et al.* A Glycomics-Based Test Predicts the Development of Hepatocellular Carcinoma in Cirrhosis. *Clin Cancer Res* **23**, 2750-2758, doi:10.1158/1078-0432.CCR-16-1500 (2017).
